# Structured medication reviews for adults with multimorbidity and polypharmacy in primary care: a systematic review

**DOI:** 10.1136/bmjopen-2026-117955

**Published:** 2026-10-22

**Authors:** Elena Lammila-Escalera, Geva Greenfield, Reham Aldakhil, Hadar Zaman, Linwei Li, Thomas Woodcock, Ana Luisa Neves, Azeem Majeed, Benedict Hayhoe

**Affiliations:** 1 Department of Primary Care and Public Health, Imperial College London, London, UK; 2 Department of Pharmacy, North Cumbria Integrated Care NHS Foundation Trust, Penrith, UK; 3 School of Pharmacy and Medical Sciences, University of Bradford, Bradford, UK

**Keywords:** Polypharmacy, Multimorbidity, Primary Care, Deprescriptions, Drug Utilization

## Abstract

**Abstract:**

**Objectives:**

Structured medication reviews (SMRs) in primary care offer a patient-centred approach to optimising medicines for adults with multimorbidity and polypharmacy, but their effect on endpoint outcomes is not yet well-established. This review summarises their impact on these outcomes and on medicines optimisation, while also highlighting common delivery methods (in-person or remote).

**Design:**

Systematic review using narrative synthesis.

**Data sources:**

Ovid MEDLINE, Ovid EMBASE, Web of Science and CINAHL via OVID were searched for studies published between January 1997 and August 2025.

**Eligibility criteria:**

Studies of adults with multimorbidity and polypharmacy receiving an SMR in primary care, compared with usual care. Randomised controlled trials, non-randomised controlled trials, cohort and cross-sectional designs were eligible.

**Data extraction and synthesis:**

Two reviewers independently screened studies; one reviewer extracted data and a second verified them. Study quality was assessed using the National Heart, Lung, and Blood Institute quality assessment tools. Findings were synthesised narratively.

**Results:**

Nine studies (8213 participants) assessed SMRs in relation to physical health (n=5), functional status (n=5), safety (n=2), healthcare utilisation (n=7), cost-effectiveness (n=3) and person-centred outcomes (n=3). Most reported no changes in physical health and falls (n=5), with no improvement in functional status (n=5). Safety outcomes showed no differences in survival or mortality (n=2). Healthcare use was largely unchanged (n=7), with only one study reporting fewer medication-related admissions. Person-centred outcomes remained unchanged (n=2). Cost outcomes were favourable, with savings (n=3) and quality-adjusted life year gains (n=2). SMRs were associated with prescription changes (n=4), reduced inappropriate prescribing (n=3) and improved adherence (n=2), although several between-group comparisons showed no significant differences. SMRs were usually delivered by general practitioners, pharmacists or multidisciplinary teams to older adults, although the mode of delivery (in-person or remote) was often unclear.

**Conclusions:**

SMRs showed little effect on endpoint outcomes, but most studies focused on prescribing rather than clinical effects, limiting this conclusion. Realising their potential requires supportive policy and collaborative practice. Unclear delivery methods hinder evaluation, and as primary care adopts digital solutions, further research on remote SMRs is needed.

**Trial registration number:**

PROSPERO CRD42023454965.

**STRENGTHS AND LIMITATIONS OF THIS STUDY**

- This review followed established systematic review methodology, using the Cochrane Handbook, Preferred Reporting Items for Systematic Reviews and Meta-Analysis (PRISMA) guidelines and a preregistered PROSPERO protocol.
- Search strategies were developed with a specialist librarian and applied to four major electronic databases.
- Inconsistent terminology for structured medication reviews required a deliberately broad search strategy, increasing screening burden and the risk of misclassification.
- Heterogeneity in interventions, populations and reported outcomes limited quantitative synthesis.

## Introduction

 Polypharmacy, which is commonly defined as the simultaneous use of five or more medications, has emerged as a significant challenge for primary care.^1–3^ It is estimated that 45% of individuals aged 65 years and older take five or more medications daily.^4^ This increase is primarily due to longer life expectancy and a higher prevalence of multiple long-term conditions, known as multimorbidity.^5^

Polypharmacy is often necessary to achieve optimal management of multiple health conditions. However, when inappropriate, it poses significant risks to patients and places considerable strain and economic burden on the healthcare systems that support them.^6^ In a study by Brekke *et al* of 85 836 older patients in primary care, 18.4% received one or more inappropriate prescriptions.^7^ Inappropriate polypharmacy can contribute to prescribing errors, adverse drug reactions and harmful drug interactions. In England, an estimated 237 million medication errors occur annually, with 38.4% occurring in primary care.^8^ This has a detrimental impact on frailty, risk of falls and mortality, which is more pronounced in older people.^9–12^ Moreover, this can increase the treatment burden for individuals, often requiring patients to spend 5–8 hours daily on health management tasks, which can negatively impact their quality of life.^13 14^ People with lower income, polypharmacy or mental health conditions were more likely to report medication management difficulties.^15^

Medication review (MR) is one intervention designed to address inappropriate polypharmacy. An MR involves thoroughly assessing an individual’s medications, considering appropriateness, dosage and potential interactions.^16^ Although MR improves medication-related outcomes such as deprescribing, it has a minimal impact on clinical outcomes.^17^ Furthermore, MRs are often conducted remotely. Remote care, delivered via telephone, video or online platforms, offers a solution for efficient access to healthcare and condition management.^18^ General practitioners and pharmacists perceive remote MRs as procedural and something to be completed quickly, despite guidelines advocating shared decision-making and, where appropriate, family/carer involvement.^19^

Therefore, structured medication reviews (SMRs) have emerged as a promising alternative to MRs.^20 21^ SMRs provide a comprehensive assessment tailored to the patient’s needs and circumstances, including holistic care elements that extend beyond MR components. While clinical pharmacists typically conduct SMRs, complex cases often require a multidisciplinary approach.

Previous systematic reviews have broadly explored pharmacist services and deprescribing practices but have not yet focused explicitly on SMRs.^17 22–24^ Research on SMRs has primarily focused on frail older adults with specific conditions, examining settings such as community, secondary and long-term care facilities.^25–29^ Despite their potential benefits, we urgently need to clarify their impact on patient health and healthcare outcomes for patients with multimorbidity and polypharmacy. This creates a gap in understanding their effects on the general primary care population and the role of remote SMR delivery in a digitalised healthcare environment.^30^

This systematic review aims to address this gap by: (1) evaluating the effectiveness of SMRs in improving outcomes for adults with multimorbidity and polypharmacy in primary care, and (2) examining medicines optimisation outcomes and trends in SMR delivery (eg, face-to-face, remote through technology).

## Methods

A systematic literature review was performed, following the recommendations of the Cochrane Handbook for Systematic Reviews and the Preferred Reporting Items for Systematic Reviews and Meta-Analysis (PRISMA) Guidelines.^31 32^ The systematic review protocol was previously published and also registered on PROSPERO (CRD42023454965).^33 34^ Deviations from the original protocol have been highlighted using a ‘*’ symbol throughout the methodology. In a change from the protocol, made before screening commenced, physical, mental, functional and health service outcomes were designated as primary outcomes and medication-related measures, including medicines optimisation, as secondary. This reflects the review’s focus on the impact of SMRs on patient health outcomes, rather than on prescribing processes, as the more meaningful test of their effectiveness.

### Eligibility criteria

The inclusion and exclusion criteria were developed using the Population, Intervention, Comparator, Outcome, and Study Designs (PICOS) framework (table 1).^35^ This systematic review focuses on assessing the effectiveness of SMRs conducted in primary care to improve outcomes for adults with multimorbidity and polypharmacy. Studies published between 1997 and 2025 were included to ensure comprehensive coverage of relevant evidence, reflecting the Royal College of Physicians’ 1997 recommendation to integrate MRs into routine practice.^36^

**Table 1:** Inclusion criteria categorised by the PICOS framework

PICOS category	Inclusion	Exclusion
Population	Adults (aged ≥18 years) with multimorbidity (≥2 chronic conditions) and polypharmacy (concurrent prescription of ≥5 regular medications).	Paediatric populations <18 years old
Intervention	An SMR or a ‘comprehensive review of a patient’s medication, considering all aspects of their health’. In primary care, SMRs may be recorded variably across systems and studies, where coding is used (eg, SNOMED CT codes). This can also distinguish them from other medication reviews. An SMR includes elements of holistic care, extending beyond an MR. Conducted in a primary care setting by a range of healthcare professionals, multidisciplinary teams or a sole clinician.	An MR is defined by evaluation of a patient’s medicines with the aim of optimising medicines use and improving health outcomes.
Comparator	The usual or standard care provided to patients with multimorbidity and polypharmacy in primary care.	No comparator of usual or standard care.
Outcomes*	Primary outcomes included physical (eg, falls), mental (eg, depression, anxiety), functional (eg, quality of life), safety (eg, mortality) and health service outcomes (eg, healthcare utilisation, cost-effectiveness), as well as quantitatively reported patient experience. Secondary outcomes: medicines optimisation-related outcomes (changes in medication dose, frequency and dosage form), medication adherence and drug safety (eg, adverse drug events).	Any other outcomes.
Study design	Randomised controlled trials, non-randomised controlled trials, cohort, cross-sectional and case-control studies. Relevant studies with experimental and observational designs that offer quantitative data, including *cost-effectiveness.	Qualitative study designs, commentaries, conference papers, case reports, reviews.

* Deviation from original protocol.

≥, the same as or more than; <, less than; MR, medication review; PICOS, Population, Intervention, Comparator, Outcome, and Study Designs; SMR, structured medication review; SNOMED CT, Systematised Nomenclature of Medicine—Clinical Terms.

### Information sources and search strategy

The databases Ovid MEDLINE, Ovid EMBASE, Web of Science and CINAHL via OVID were searched from January 1997 to August 2025. Reference lists of included studies were also hand-searched to identify additional eligible studies. Automation tools supported the search design, including the ‘Word Frequency Analyser’, the ‘Polyglot Search Translator’ and the ‘SearchRefinery’.^37 38^ Additionally, a specialist librarian with expertise in evidence synthesis collaborated on the development of the search strategy (online supplemental appendix A). To account for variations in terminology and cultural differences across countries, the strategy includes known synonyms for SMR, such as drug utilisation review.

### Selection process and data collection process

Following removal of duplicates using the specialist screening platform ‘Covidence’, two reviewers (EL-E and RA) independently screened records in two stages: title and abstract, followed by full text. Before title and abstract screening, the reviewers completed a calibration exercise on an initial sample of 100 records to confirm consistent application of the eligibility criteria. Screening proceeded only once at least 90% inter-reviewer agreement was reached. All records were subsequently screened independently and in duplicate at both stages. Disagreements were resolved through discussion between the two reviewers and consensus was reached in all cases. The wider team was consulted where clarification of the eligibility criteria was required.

### Data extraction process

Data were extracted by one reviewer (EL-E) using an extraction form and independently verified by a second (RA), with any discrepancies resolved through discussion. Extracted fields included study identifier, study design and duration, participant characteristics, outcome measures, SMR description and mode of delivery.

### Risk of bias assessment

EL-E assessed the risk of bias in included studies. For observational cohort studies, cross-sectional studies, pre-post intervention studies and randomised controlled trials, the risk of bias was assessed using the National Heart, Lung, and Blood Institute Quality Assessment Tool.^39^ *For cost-effectiveness analyses, the Consensus on Health Economic Criteria list was used.^40^ RA then verified this risk-of-bias assessment.

### Synthesis methods

Given the expected heterogeneity of SMR interventions and the complexity of multimorbidity, meta-analysis was not feasible. Instead, a narrative synthesis was used to summarise the evidence on the impact of SMRs.

### Patient and public involvemen

Patients and the public were not directly involved in the design or conduct of this study, which synthesised existing peer-reviewed research rather than collecting primary data. The review nonetheless prioritised patient-relevant outcomes, including patient experience and health outcomes, when evaluating the effectiveness of SMRs.

## Results

### Study selection

Searches identified 3098 records (figure 1). Of the 59 studies selected for full-text screening, 47 were excluded due to differences in patient population, intervention, outcome focus and setting. Twelve studies were consequently identified as eligible for inclusion in this review.^41–52^ Three of these were cost-effectiveness analyses,^50–52^ conducted alongside three of the included intervention studies,^41 44 48^ which shared identical populations and settings. To avoid duplication, each cost-effectiveness analysis was merged with its corresponding intervention study and reported as a single study (online supplemental appendix B). Consequently, nine studies are presented in this review.^41–49^

**Figure 1:**
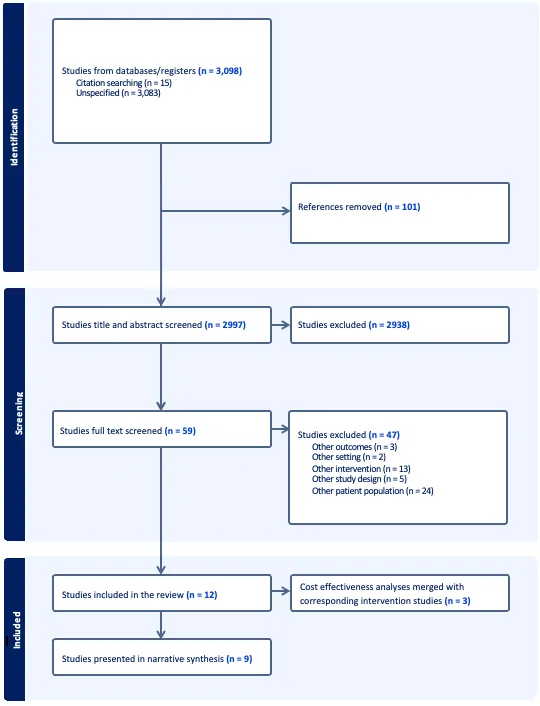
Preferred Reporting Items for Systematic Reviews and Meta-Analysis (PRISMA) flow chart.

### Study characteristics

The nine included studies (online supplemental appendix B) were published between 2013 and 2025 across Switzerland,^41^ the Netherlands,^42^ Sweden,^43^ Germany,^45^ Ireland,^44 48^ Australia,^47^ Italy,^49^ and one multinational study conducted across four countries.^46^ They comprised six randomised controlled trials,^41 43–46 49^ one non-randomised open controlled study^42^ and two prospective pre-post intervention studies,^47 48^ alongside the three linked economic evaluations.^50–52^ In total, 8213 participants were included, with mean or median ages ranging from 71.7^47^ to 81.5 years^46^. Patients had a median or mean of approximately 3.4^43^ to 9.5^46^ chronic conditions, with one study instead characterising comorbidity using the Charlson Comorbidity Index.^45^ The length of follow-up varied from under 12 weeks^48^ to 24 months.^46 49^ The studies recruited participants with a broad combination of comorbidities and polypharmacy.

### Risk of bias

Two studies were rated as good quality^41 45^, three as fair^44 46 49^ and four as poor^42 43 47 48^ (online supplemental appendix C). Lower ratings were driven by selection and recruitment bias,^48^ baseline imbalances^43 46 49^ and attrition or short follow-ups.^42 48 49^ For cost-effectiveness, one study was rated good,^50^ another fair^51^ and the third poor^52^ due to its single-arm design, absence of a controlled counterfactual and reliance on assumption-based costing.

### SMR delivery

All studies examined SMRs in a primary care general practice setting or in the patient’s home (table 2). In seven studies, the patient selection criteria for the SMR specified that participants be over 60 years old,^41–46 49^ whereas one study included individuals over 18 years old.^47^ Most SMRs involved general practitioners,^41 44–46^ a pharmacist or both.^42 47^ A few included the involvement of a nurse,^42^ a healthcare assistant^45^ or an internal medicine specialist.^49^

**Table 2:** SMR description and components

Author(s)	SMR components				
	Description	Patient selection criteria	Setting	HCP conducting the SMR	Number of contacts
Face-to-face delivery					
McCarthy *et al*^44 50^	GP-led SPPiRE medication review.	Age ≥65, with ≥15 prescribed medications.	General practice.	General practitioners.	1
Tan *et al*^47^	Pharmacist-led multidisciplinary medication review.	Age >18 with >5 medications that require therapeutic drug monitoring, using medicines for >3 medical conditions and recent unplanned healthcare utilisation.	Primary care clinic or home.	Pharmacist, general practitioner.	2
Both remote and face-to-face delivery					
Kinahan *et al*^48 52^	Person-centred medication review	Age ≥50 years living in a residential care setting, ≥10 regular prescribed medications, a greater risk of medicines-related harm or adults of any age approaching the end of their life due to any cause.	General practice.	Pharmacist	2
Delivery mode not reported					
Jungo *et al*^41 51^	Six-step medication review using STRIPA (web-based electronic clinical decision support system).	Age ≥65, ≥5 long-term medications (≥90 days), ≥3 chronic conditions.	General practice.	General practitioner.	1
Leendertse *et al*^42^	Multicomponent pharmaceutical care intervention.	Age ≥65, ≥5 chronically prescribed drugs with ≥1 refill rate of <80% or >120%. ≥1 drug from the Anatomical Therapeutic Chemical Class A or Class B.	General practice.	General practitioner, pharmacist and primary care practice nurse.	>3
Lenander *et al*^43^	Preparatory structured pharmaceutical advice.	Age ≥65 with ≥5 medications who were already scheduled for an appointment with a GP.	General practice.	Geriatric pharmacist.	1
Mahlknecht *et al*^49^	Interdisciplinary review.	Age ≥75 with ≥8 prescribed medications.	Primary care.	Specialist in internal medicine, a clinical pharmacologist, an evidence-based medicine expert, general practitioner.	1
Muth *et al*^45^	Prioritising multimedication in multimorbidity multicomponent review.	Aged ≥60, ≥3 chronic conditions under pharmacological treatment, ≥5 long-term prescriptions of drugs with systemic effects.	General practice.	General practitioner, healthcare assistant.	1
Rieckert *et al*^46^	Electronic decision support tool comprising a comprehensive drug review to support general practitioners.	Age ≥75, with ≥8 prescribed medications.	General practice.	General practitioner.	≥4

GP, general practitioner; HCP, healthcare professional; SMR, structured medication review; SPPiRE, Supporting Prescribing in Older Adults with Multimorbidity in Irish Primary Care; STRIPA, Systematic Tool to Reduce Inappropriate Prescribing Assistant .

Two studies examined the SMR within a multidisciplinary approach,^47 49^ while two were supported by technology.^41 46^ The number of contacts varied from one^41 43–45 49^ to more than three.^42 46^ Two studies reported delivering SMRs face-to-face^44 47^, while one study^48^ noted that although SMRs started as phone sessions due to COVID-19, they later shifted to face-to-face delivery. Six studies did not specify the delivery method,^41–43 45 46 49^ and no studies reported exclusively remote delivery via technology.

### Outcomes

Of the nine studies, five reported physical health outcomes such as falls or self-rated health,^41 43 45 47 49^ five reported functional health outcomes such as health-related quality of life,^41 42 45 48 49^ two reported safety outcomes such as mortality and survival,^42 46^ three reported cost-effectiveness outcomes,^50–52^ seven reported health utilisation outcomes such as hospital admissions and general practitioner visits^42–47 49^ and three reported patient-centred outcomes such as understanding of medicines (table 3).^44 45 48^ Outcomes exploring medicines optimisation measures such as prescription changes, medication adherence and drug safety were also widely reported (n=9),^41–49^ (table 4).

**Table 3:** Primary outcomes

Outcome	Findings
Physical health (n=5)	One study found fewer falls in the intervention group but no difference in fractures,^49^ while another found no difference in falls.^41^ A third observed no difference in pain scores.^45^ Additionally, one reported stable self-rated health in the intervention group but a significant decline in the control group,^43^ whereas another found no difference.^47^
Functional health (n=5)	Four studies^41 42 45 48^ found no differences or changes in quality-of-life scores, one of which also reported no difference in expected life duration^45^ and another no difference in health-related quality of life or cognitive function.^49^
Safety (n=2)	Two studies^42 46^ found no significant differences in survival or mortality between groups.
Health service utilisation (n=7)	One study^42^ reported fewer medication-related hospital admissions in the intervention group, while two others^43 49^ found no reduction. Additionally, one study saw no difference in unplanned healthcare visits^46^ and another found no change in overall health service use.^47^ One study^44^ found no effect on healthcare utilisation, with general practice visits falling and telephone consultations rising in both groups, while another reported no reduction in primary care use.^43^ Two studies showed no significant differences in length of stay.^43 45^
Cost-effectiveness (n=3)	Three studies reported cost-savings^50–52^, two of which also reported QALY gains.^50 51^ One demonstrated significant savings of CHF 1857 per patient and a QALY gain of 0.026, with stronger effects in men, those aged 65–75 and those aged over 85 years.^51^ One study estimated annual drug cost savings of €376 per patient, equating to approximately €178 000 net savings per pharmacist per year.^52^ Another study reported a mean cost reduction of €410 and QALY gains of 0.014, with a 99% probability of cost-effectiveness despite non-significant differences.^50^
Person-centred outcomes (n=3)	One study reported that 87.7% of participants felt the review improved their understanding of medicines and 30.5% that it reduced the negative impact of medicines on their daily activities.^48^ Two further studies assessed patient-reported constructs but found no significant between-group effects.^44 45^ Specifically, one reported no difference in treatment burden or attitudes to deprescribing^44^ while the other reported no difference in satisfaction with shared decision-making or beliefs about medicines.^45^

%, percentage; €, Euro; CHF, Swiss Franc; n, number; QALY, Quality-Adjusted Life Year.

**Table 4:** Secondary outcomes

Outcome	Findings
Prescription changes (n=5)	Deprescribing occurred across studies. In one, more medications were stopped and odds of being prescribed ≥15 medicines were lower in the intervention group.^44^ Two trials reported significant between-group reductions in the number of medicines^43 46^ one of which also found more drug adjustments or eliminations in the intervention group.^46^ One study reported a mean reduction of 1.5 per patient.^48^ Another found 37.2% of patients had at least one drug stopped, but with no significant difference between the groups and that 76.5% of patients received recommendations for discontinuation.^49^
Inappropriate prescriptions (n=5)	The intervention reduced potentially inappropriate prescriptions,^46^ with 70.7% of 1056 high risk prescribing indicators resolved.^48^ Another study identified 15.8% of prescriptions as inappropriate.^49^ In addition, one study noted a mean reduction of 17.3 PC-MAI score points per patient.^48^ However, one study found no consistent change in PIP odds^44^ and another reported a small, non-significant effect on medication appropriateness index scores.^45^
Medication adherence (n=5)	Two studies^43 47^ found significant improvements in medication adherence, while another^42^ found no difference between groups. A further trial^45^ observed small, but non-significant changes in adherence, while in another study 36.9% of participants reported an improved ability to take medicines correctly following the intervention.^48^
Drug safety (n=6)	One study^47^ observed a significant reduction in medication-related problems, while another^41^ reported fewer safety events in the intervention group. 63.1% of participants from one study reported that side effects improved for them following the intervention.^48^ However, three studies^42 43 46^ found no significant differences in adverse drug events or dosage issues.

%, percentage; n, number; PC-MAI, Person-Centred Medication Appropriateness Index; PIP, potentially inappropriate prescribing.

## Discussion

### Main findings

Across the nine included studies, SMRs did not consistently improve the physical health, functional status or safety of patients with multimorbidity and polypharmacy in primary care. The majority, however, were designed to assess prescribing-related outcomes rather than broader measures, and several were of fair or poor methodological quality; thus, the strength of this conclusion may be limited. Although SMR interventions have been reported as cost-effective, findings on healthcare utilisation were largely unchanged. Most studies reported no reduction in use after SMR, although one noted fewer medication-related admissions. Person-centred outcomes were rarely measured, and where treatment burden, shared decision-making and beliefs about medicines were assessed, none improved. However, SMRs led to changes in prescribing practices, including deprescribing and a reduction in inappropriate prescriptions. Hence, while SMRs did not consistently improve broader health outcomes, their ability to optimise prescribing suggests a useful role for them in medicines optimisation.

Reviews were often conducted by small teams of primary healthcare professionals (including general practitioners, pharmacists or multidisciplinary teams). In general, these were performed in general practice or at the home of the patient, with patients selected for SMRs generally older. Although the small number of studies and their heterogeneity preclude formal comparison, improvements clustered by what each study measured rather than by how the review was configured: gains in medicines-optimisation outcomes appeared across GP-led, pharmacist-led and multidisciplinary reviews and in studies with and without decision-support tools, whereas clinical endpoints showed little consistent change in any configuration. Improvements were therefore concentrated in the prescribing changes SMRs directly enact, rather than the broader health outcomes they are ultimately intended to improve. However, the specific delivery methods of the SMRs were not clearly defined in the studies reviewed, limiting the review’s ability to draw generalisable conclusions about the effectiveness of different SMR delivery approaches.

### Strengths and limitations

To our knowledge, this systematic review is the first to evaluate the effectiveness of SMRs on a range of outcomes for patients with multimorbidity and polypharmacy. A key strength of this review is its use of automation tools, which have improved search and screening efficiency while streamlining the development of search strings. Additionally, the inclusive approach addresses the historical underrepresentation of multimorbidity in research, contributing to clinical guidance for managing its challenges.

The threshold for defining polypharmacy varies across the literature. While this review adopted a cut-off of five or greater for inclusion, other studies have used thresholds as low as two or as high as eleven.^3^ As a result, our definition may have excluded studies that use alternative definitions. In addition, despite a clear definition of SMRs, distinguishing them from general MRs was challenging because the literature uses inconsistent terminology, with SMRs and MRs often used interchangeably. This issue was further complicated by the emergence of alternative terms for SMRs, such as ‘comprehensive’ medication and ‘medication management’ reviews, highlighting the lack of consensus on terminology. Nevertheless, adherence to National Institute for Health and Care Excellence guidance, the Pharmaceutical Care Network Europe definition of MRs, and prioritising studies that incorporate shared decision-making enabled the review to effectively differentiate SMRs from MRs.

Another limitation is the difficulty in determining the mode of delivery for SMRs, likely due to limited literature and the application of a strict SMR definition. The complexity of the assessed interventions also posed a challenge, with multiple components such as MR, holistic clinical assessment, shared decision-making and follow-up monitoring rather than a single action. While these components underscore the difficulties of addressing potentially inappropriate prescribing in patients with multimorbidity, they also limit the ability to attribute improvements to specific components, complicating the identification of which components had the greatest impact on outcomes. The diversity in study designs, populations, interventions and outcome measures among the included studies prevented meta-analysis and required a narrative synthesis. Although this approach enabled the consideration of a wide range of outcomes, it also restricts the capacity to quantify the overall impact of SMRs or to perform statistical comparisons across studies. Another limitation of the evidence base is that few studies measured person-centred outcomes, which are central to the justification for SMRs. This restricts our ability to assess whether these interventions effectively meet their intended patient-orientated goals. Study quality was variable, with several studies rated fair or poor, reflecting common methodological constraints such as small samples, recruitment and selection biases and baseline imbalances. The absence of consistent improvement in clinical outcomes should therefore be interpreted with corresponding caution.

### Comparison with existing literature

As this is the first systematic review of SMRs, findings are compared with broader MR and deprescribing literature. Our findings are consistent with evidence that MRs can reduce prescription volume, identify inappropriate polypharmacy and lower the risk of adverse drug events.^17 22 53^ However, MRs do not consistently improve endpoint outcomes such as falls, cognition, quality of life or hospital admissions.^17 22 53^ Where review-level benefit does appear, it is largely confined to disease-specific markers rather than the broader endpoints examined here.^54^ Prior research also suggests that MRs, like SMRs included in this review, are associated with reduced medication expenditure.^55^

The complexity of SMR interventions observed in this review reflects wider evidence that effective deprescribing through MRs requires multifaceted approaches.^23^ Collaboration between pharmacists and physicians is consistently highlighted as essential to optimise medication use and reduce inappropriate prescribing.^24^ Multidisciplinary team models have also been shown to reduce hospital visits and improve quality of life, reinforcing the pharmacist’s role within general practice-delivered SMR services.^56^

A prior scoping review of telehealth MRs similarly highlighted the lack of evidence regarding remote delivery, concluding that these limited data cannot reliably guide practice, which aligns with the findings of this review.^57^ Additionally, only two interventions incorporated technology-assisted decision-making, although computerised decision-support tools are used for prescribing in primary care. However, these tools have not been shown to improve health outcomes.^58^

### Implications for research

Further research should evaluate whether SMRs achieve the holistic health benefits they are intended to deliver, rather than focusing predominantly on prescribing outcomes. The limited number of studies assessing and powered to evaluate outcomes beyond medicines optimisation highlights the importance of comprehensive evaluations of SMRs. If SMRs are intended as holistic health assessments, their impact on physical, mental and functional health, as well as patient-reported outcomes, requires more rigorous investigation. The current heterogeneity of interventions also hinders meaningful comparison, underscoring the need for greater standardisation of procedure, with adaptable components tailored to local patient needs and resources.

Equity considerations should be a priority. None of the studies reported outcomes by socio-economic status, ethnicity or language proficiency. Given the strong association between deprivation, multimorbidity and polypharmacy, it is essential to identify which patient groups benefit most from SMRs. Future research should also examine how SMRs address the needs of patients with language barriers, cognitive impairment or disabilities, as these reviews are designed to facilitate communication and shared decision-making between patients and their clinicians. Although SMRs are intended to incorporate patients’ social history and lifestyle factors, such as alcohol use, few of the included studies reported doing so in practice. Making fuller use of this holistic scope could strengthen the alignment of SMRs with wider initiatives such as social prescribing.

Improved reporting of intervention components is also critical to enhance replicability, particularly for remote delivery. While remote SMRs appear feasible, their impact on patient outcomes remains unclear.^57^ Research should examine whether shared decision-making and person-centred care can be effectively achieved in remote settings and whether patients are satisfied with this approach. Testing structured models for remote consultations, as well as evaluating approaches to remote collaboration in interdisciplinary teams, will be important.

### Implications for practice and policy

This review provides evidence to guide the implementation of SMRs for patients with multimorbidity and polypharmacy, a particularly complex population.^30^ For SMRs to achieve impact, they must be delivered through collaborative frameworks underpinned by clear policy, leadership and evaluation.^59^ Guidance should clearly define what separates SMRs from general MRs, including where they are delivered, how much patients participate, and the role of informal caregivers. Rather than focusing on which professional delivers the review, practice and policy should prioritise interdisciplinary collaboration. Formal agreements with defined roles, supported by multidisciplinary team meetings already used in multimorbidity care, offer a model for coordinated delivery.^60^ Digital technologies also offer an opportunity to facilitate communication across dispersed teams.

Effective implementation also requires clarity on the tools and expertise needed. Clinicians should receive ongoing training in person-centred consultation skills, enabling stronger patient-provider relationships that foster trust, shared decision-making and opportunities for patient education. At the same time, systemic barriers, such as limited consultation time or workload, may impact the quality of SMRs delivered. Organisational support must address these factors, without which the potential of SMRs will remain unrealised.

## Conclusion

SMRs were introduced as a person-centred response to the challenges of polypharmacy, intended to provide a holistic assessment of patients’ health and medications. This systematic review found that while SMRs led to improved prescribing practices, they did not consistently enhance endpoint outcomes or reduce healthcare utilisation in patients with multimorbidity and polypharmacy. This should be understood in the context of an evidence base that is small, diverse and primarily focused on detecting prescribing changes rather than health outcomes, reflecting both the constraints of the available evidence and potentially the intervention itself. This apparent gap between intention and impact warrants attention, particularly given the comprehensive nature of the ideal SMR model. A key limitation was the lack of clarity around how these SMR interventions were delivered, making it challenging to identify the most effective approaches. Despite these challenges, SMRs offer a promising framework for improving prescribing quality, though their broader person-centred benefits remain to be demonstrated. To fulfil their potential, SMRs must be underpinned by clear policy guidance, embedded within collaborative models of care and supported by adequate training and structural resources. Clinicians need the time and support to deliver meaningful reviews so patients can truly benefit from the intended value of SMRs.

## Supplementary material

10.1136/bmjopen-2026-117955 online supplemental file 1

## Data Availability

All data relevant to the study are included in the article or uploaded as supplementary information.
