# Supplementary material for: Structured medication reviews for adults with multimorbidity and polypharmacy in primary care: a systematic review
Source: BMJ Open. 2026 Oct 22;16(10):e117955. doi: 10.1136/bmjopen-2026-117955 (PMC13691817; doi:10.1136/bmjopen-2026-117955)
Supplement: Supplementary file 1 [file bmjopen-2026-117955.reviewer_comments.pdf]

PEER REVIEW HISTORY

BMJ Open publishes all reviews undertaken for accepted manuscripts. Reviewers are asked to complete a checklist review form and are provided with free text boxes to elaborate on their assessment. These free text comments are reproduced below.

ARTICLE DETAILS

Title (Provisional)

Structured medication reviews for adults with multimorbidity and polypharmacy in primary care: a systematic review

Authors

Lammila-Escalera, Elena; Greenfield, Geva; Aldakhil, Reham; Zaman, Hadar; Li, Linwei; Woodcock, Thomas; Neves, Ana Luisa; Majeed, Azeem; Hayhoe, Benedict

---

VERSION 1 - REVIEW

**Reviewer**	**1**
**Name**	**Gangannagaripalli, Jaheeda**
**Affiliation**	**The University of Manchester, NIHR ARC GM /Healthy Ageing research group, School of Health Sciences**
**Date**	**23-Jun-2026**
**COI**	**None**

This is an important and timely study, as structured medication reviews (SMRs) in primary care and their outcomes have not yet been fully evaluated. The findings of this review will inform future research and support the implementation of SMRs in primary care settings. Please find my suggestions below, which may help improve the draft.

Abstract:

Line 7: suggest adding adverse drug events.

Line 20: Please provide the host for CINAHL, if available.

Line 29: For “in (n=1)”, please add the outcome.

Line 37: “Primary care teams including” — please clarify who the team members are.

Strengths and limitations

Line 16: suggest adding meta-analysis.

Introduction:

Lines 55–57: Please add references for community, secondary, and long-term care facilities.

Table 1: Outcomes domain: Why are adverse drug events not included?

Information sources and search strategy:

Line 49: Please add the host for the CINAHL database, if available.

Selection process and data collection process: Many details are missing. There is no indication of pilot screening or confirmation of eligibility criteria among the reviewers for both title-abstract and full-text screening. There is also no clear distinction between title-abstract and full-text screening. How were discrepancies resolved? Was a third reviewer involved? If these steps were not followed, please add this to the limitations.

Data extraction process: There is no mention of independent duplicate screening or piloting. Again, please add this as a limitation.

PRISMA flow chart: There appears to be an inconsistency between the text and the chart regarding the number of studies finally included. Please correct this.

Results:

Study characteristics: Eight countries are mentioned; please specify which countries were included. Some information is incomplete. Please summarise the types of studies included, expand on the types of comorbidities, and specify the mean ages of the study populations. Please also include the types and number of studies included.

Risk of bias: Line 55: Please briefly summarise which studies reported these limitations.

Outcomes: Please include details of what the outcomes are. I do not think it is sufficient to mention only physical health outcomes. I suggest adding more detail on what these outcomes are. The same applies to all other outcomes. I do not think referring only to the table of outcomes would be sufficient.

Discussion: Please briefly summarise the results here, including how many studies were included and what they focused on.

Strengths and limitations:

Line 56: Could you please expand on the inclusive approach?

Page 14, line 26: Specific components — what are these components?

Page 14, lines 51–54: Please provide references.

Page 14, line 57: The point about further research is unclear. Could you please clarify this?

Page 15, line 15: suggest adding patients with cognitive impairment and disabilities.

Page 15, line 19: From my understanding, SMRs take social history into consideration.

Comparison with the literature: Are there no studies specifically focused on SMRs that could be used for comparison?

**Reviewer**	**2**
**Name**	**Divac , Nevena**
**Affiliation**	**University of Belgrade**
**Date**	**30-Jul-2026**
**COI**	**None**

This review examines an important question concerning the effectiveness of structured medication reviews for adults of different age categories with polypharmacy in primary care. By synthesising evidence from a limited but growing body of literature, the authors provide a useful overview of intervention characteristics and outcomes. The manuscript is generally well presented and addresses a topic with clear implications for clinical practice and healthcare policy. Nevertheless, several methodological and interpretative issues require clarification before the findings can be published.

1. The included interventions vary considerably with respect to professional involvement (GPs, pharmacists, multidisciplinary teams), use of decision-support tools, intervention intensity, follow-up duration, and care settings. Although the authors appropriately chose narrative synthesis because of heterogeneity, the discussion would benefit from a more structured exploration of whether certain intervention characteristics were associated with more favorable outcomes. This would help readers understand which components of SMRs may be most promising.

2. SMRs are presented as person-centred interventions, yet patient experience and patient-reported outcomes receive comparatively little attention in the Results and Discussion sections. The manuscript would benefit from a more explicit synthesis of outcomes related to patient satisfaction, understanding of medicines, shared decision-making, and treatment burden, which are central to the rationale for implementing SMRs. If those are not available, that should be emphasized in the Limitations section.

3. The conclusions should be interpreted with greater caution. Although the review did not identify consistent improvements in clinical outcomes, the evidence base is limited, heterogeneous and includes several studies judged to be of fair or poor quality. In addition, many of the included studies appear to have been primarily designed to assess prescribing-related outcomes rather than patient-centered clinical endpoints. The authors should therefore discuss more explicitly how these limitations may influence the interpretation of

the findings and avoid conclusions that could be interpreted as evidence of lack of effectiveness.

VERSION 1 - AUTHOR RESPONSE

Reviewer Comments	Author Response
Reviewer 1	
Abstract	
Line 7: suggest adding adverse drug events.	Thank you for the suggestion. We have medicine-optimisation results specified in the Objectives subsection of the abstract but due to a limited word count we were unable to distil the outcome umbrella further without losing vital detail from other subsections.
Line 20: Please provide the host for CINAHL, if available.	We have added the host for CINAHL to the new Data Sources subsection of the abstract. The revised subsection now reads, "*Data Sources: Ovid MEDLINE, Ovid EMBASE, Web of Science, and CINAHL via OVID were searched between January 1997 and August 2025.*"
Line 29: For "in (n=1)", please add the outcome.	Thank you for your comment, after restructuring the abstract and to account for the wordcount, the results have been rephrased and the passage reads as follows: "*Nine studies (8,213 participants) assessed SMRs in relation to physical health (n = 5), functional status (n = 5), safety (n = 2), healthcare utilisation (n = 7), cost-effectiveness (n = 3), and person-centred outcomes (n = 3). Most reported no changes in physical health, falls, or quality of life (n = 5), and no improvement in functional status (n = 5). Safety outcomes showed no differences in survival or mortality (n = 2). Healthcare use was largely unchanged (n = 7), with only one study reporting fewer medication-related admissions. Person-centred outcomes remained unchanged (n = 2). Cost outcomes were favourable, with savings (n = 3) and QALY gains (n = 2). SMRs were associated with prescription changes (n=4), reduced inappropriate prescribing (n=3), and improved adherence (n=2), though most between-group comparisons showed no significant differences (n=5). Delivery was usually by general practitioners, pharmacists or multidisciplinary teams to older adults, though the delivery method (in-person or remote) was often unclear.*"
Line 37: "Primary care teams including" — please clarify who the team members are.	Thank you for your suggestion, we have now added more detail to the abstract, clarifying who the primary care team members are. The sentence now reads as follows, "*Delivery was usually by general practitioners, pharmacists or multidisciplinary teams to older adults, though the delivery method (in-person or remote) was often unclear.*"
Introduction	
Lines 55–57: Please add references for community, secondary, and long-term care facilities.	Thank you for identifying this. We have now added references the last paragraph of the Introduction to ensure we substantiate that SMRs have been studies across

	community, secondary and long-term care settings. These include, *Khera at al 2019, Klopotowska et al 2022, Mahlknect et al 2019, Oonk et al 2024*. The sentence reads as follows, "*Research on SMRs has primarily focused on frail older adults with specific conditions, examining settings such as community, secondary, and long-term care facilities.*[25–29]"
Methodology	
Line 49: Please add the host for the CINAHL database, if available.	For the Information sources and search strategy subsection of the methods, we have now added in the host for CINAHL. The amended sentence now reads, "*The databases Ovid MEDLINE, Ovid EMBASE, Web of Science, and CINAHL via OVID were searched from January 1997 to August 2025*."
Selection process and data collection process: Many details are missing. There is no indication of pilot screening or confirmation of eligibility criteria among the reviewers for both title-abstract and full-text screening. There is also no clear distinction between title-abstract and full-text screening. How were discrepancies resolved?<br>Was a third reviewer involved? If these steps were not followed, please add this to the limitations.	Thank you for highlighting this and we agree, the previous version was light on details that would be valuable to the reader to know. We have since amended the Selection process and data collection process subsection of the methods and it now reads '*Following removal of duplicates using the specialist screening platform 'Covidence', two reviewers (ELE and RA) independently screened records in two stages: title and abstract, followed by full text. Before title and abstract screening, the reviewers completed a calibration exercise on an initial sample of 100 records to confirm consistent application of the eligibility criteria. Screening proceeded only once at least 90% inter-reviewer agreement was reached. All records were subsequently screened independently and in duplicate at both stages. Disagreements were resolved through discussion between the two reviewers and consensus was reached in all cases. The wider team was consulted where clarification of the eligibility criteria was required*.'
Data extraction process: There is no mention of independent duplicate screening or piloting. Again, please add this as a limitation.	As with the above, we agree the subsection was short and lacking in detail. We have now revised the Data extraction process subsection of the methods, and it now reads, "*Data were extracted by one reviewer (ELE) using an extraction form and independently verified by a second (RA), with any discrepancies resolved through discussion. Extracted fields included study identifier, study design and duration, participant characteristics, outcome measures, SMR description, and mode of delivery.*"
Results	
PRISMA flow chart: There appears to be an inconsistency between the text and the chart regarding the number of studies finally included. Please correct this.	Thank you for bringing this to our attention. We had not included the detail on how we emerged with 9 included studies, instead of 12. The search initially identified 12 studies eligible for inclusion, however three of these were cost-effectiveness analyses which were conducted alongside included intervention studies. To avoid duplication, each cost-effectiveness analysis was merged with its corresponding intervention study and reported as a single study. We explain this in the Study selection subsection of the results but neglected to add this detail to

	the PRISMA diagram. However, the diagram (Figure 1) has since been updated and now includes the number of studies presented in narrative synthesis as n=9.
Study characteristics: Eight countries are mentioned; please specify which countries were included. Some information is incomplete. Please summarise the types of studies included, expand on the types of comorbidities, and specify the mean ages of the study populations. Please also include the types and number of studies included.	We have now expanded the Study characteristics subsection of the results to include further detail, which was earlier confined to the only Appendices. This revised section now reads: "*The nine included studies (Appendix B) were conducted between 2013 and 2025 across Switzerland[40], the Netherlands,[41] Sweden[42], Germany[44], Ireland[43,47], Australia[46], Italy[48], and one multinational study conducted across four countries[45]. They comprised seven randomised controlled trials.[40,42–45,48], one non-randomised open controlled study[41], and two prospective pre-post intervention studies[46,47], alongside the three linked economic evaluations[49–51]. In total, 8,213 participants were included, with mean or median ages ranging from 71.7[46] to 81.5 years[45]. Patients had a median or mean of approximately five[42] to 9.5[45] chronic conditions, with one study instead characterising comorbidity by the Charlson Comorbidity Index[44]. The frequency of follow-ups varied from under 12 weeks[46] to 24 months.[45,48] The studies recruited participants with a broad combination of comorbidities and polypharmacy.*"
Risk of bias: Line 55: Please briefly summarise which studies reported these limitations.	Thank you. The Risk of bias subsection of the results has now been revised and more detail regarding which studies refer to specific limitations have been added in as citations. The subsection now reads as follows, "*Two studies were rated as good quality, three as fair,43,45,48 and four as poor41,42,46,47 (Appendix C). Lower ratings were driven by selection and recruitment bias,47 baseline imbalances,42,45,48 and attrition or short follow-ups41,47,48. For cost-effectiveness, one study was rated good,49 another fair,50 and the third poor51 due to its single-arm design, absence of a controlled counterfactual, and reliance on assumption-based costing.*"
Outcomes: Please include details of what the outcomes are. I do not think it is sufficient to mention only physical health outcomes. I suggest adding more detail on what these outcomes are. The same applies to all other outcomes. I do not think referring only to the table of outcomes would be sufficient.	Thank you for your comment. We have added more detail regarding the examples of each outcome to the text before the outcome tables. The Outcomes subsection of the results now reads, "*Of the nine studies, five reported physical health outcomes such as falls or self-rated health,[40,42,44,46,48] five reported functional health outcomes such as health-related quality of life,[40,41,44,47,48] two reported safety outcomes such as mortality and survival ,[41,44,45] three reported cost-effectiveness outcomes,[49–51] seven reported health utilisation outcomes such as hospital admissions and general practitioner visits,[40–46] and three reported patient-centred outcomes such as understanding of medicines (Table 2). Outcomes exploring medicines optimisation measures such as prescription changes, medication adherence and drug safety were also widely reported (n=9),[40–48] (Table 3).*"
Discussion	

Please briefly summarise the results here, including how many studies were included and what they focused on.	Thank you, we have now added more detail to the introductory sentences of the Main findings subsection of the discussion, summarising how many studies were included and their primary focus. These two sentence now read, "*Across the nine included studies, SMRs did not consistently improve the physical health, functional status, or safety of patients with multimorbidity and polypharmacy in primary care. The majority, however, were designed to assess prescribing-related outcomes rather than broader measures, and several were of fair or poor methodological quality; thus, the strength of this conclusion may be limited.*"
Strengths and Limitations	
Line 16: suggest adding meta-analysis.	Thank you for the suggestion. We have now added some sentences to the Strengths and limitations subsection of the discussion detailing why a meta-analysis was precluded and also why this limits the conclusions we can draw. These have been added to the end of third paragraph and now read: "*The diversity in study designs, populations, interventions, and outcome measures among the included studies prevented meta-analysis and required a narrative synthesis. Although this approach enabled the consideration of a wide range of outcomes, it also restricts the capacity to quantify the overall impact of SMRs or to perform statistical comparisons across studies*."
Page 14, line 26: Specific components — what are these components?	Thank you and we agree that this was unclear. The sentence in the 3rd paragraph of the Strengths and limitations subsection of the Discussion now reads: "*The complexity of the assessed interventions also posed a challenge, with multiple components such as medication review, holistic clinical assessment, shared decision-making and follow-up monitoring rather than a single action*."
Page 14, lines 51–54: Please provide references.	We thank the reviewer for identifying this. We have added the supporting references to this sentence which sits in the first paragraph of the Comparison to existing literature subsection of the discussion. The statement that medication reviews reduce prescription volume, identify inappropriate polypharmacy and lower the risk of adverse drug events is now supported by the systematic reviews and meta-analyses of Huiskes *et al*, Dills *et al* and Holland *et al*. The sentence now reads, "*This review is consistent with this evidence that MRs can reduce prescription volume, identify inappropriate polypharmacy and lower the risk of adverse drug events.*[17,22,57]"
Page 14, line 57: The point about further research is unclear. Could you please clarify this?	Thank you for highlighting this and we agree that the sentence was unclear. We have since refined the focus of the sentence so that it points to a tangible aspect. The introductory sentence for the Implications for research subsection of the discussion now reads, "*Further research should evaluate whether SMRs achieve the holistic health benefits they are intended to deliver,*

	rather than focusing predominantly on prescribing outcomes."
Page 15, line 15: suggest adding patients with cognitive impairment and disabilities.	Thank you for suggestion, we have added these other patient groups to the 2nd paragraph of the Implications for research subsection of the discussion. The sentence now reads, "*Future research should also examine how SMRs address the needs of patients with language barriers, cognitive impairment or disabilities, as these reviews are designed to facilitate communication and shared decision-making between patients and their clinicians*."
Page 15, line 19: From my understanding, SMRs take social history into consideration.	Thank you and yes, they do. However, few of the included studies reported taking in patients' social history in practice so our recommendation would ensure that clinicians use this component of SMR to its full potential. However, we understand that this is not clear from our previous text. Consequently, we have now clarified the end of the second paragraph of the Implications for research subsection of the discussion to ensure that the text reflects this point. The revised text now reads, "*Although SMRs are intended to incorporate patients' social history and lifestyle factors, such as alcohol use, few of the included studies reported doing so in practice. Making fuller use of this holistic scope could strengthen the alignment of SMRs with wider initiatives such as social prescribing*."
Comparison with the literature: Are there no studies specifically focused on SMRs that could be used for comparison?	There are studies that explore SMRs, however all of which are directly relevant to our study within our search timeframe are included in this systematic review. However, as this is a no systematic reviews of literature, we have focused on comparing our results to similar systematic reviews. Unfortunately, to our knowledge, this is the first systematic review focusing on SMRs for this patient population in this setting. So, SMR specific systematic reviews that are directly applicable to ours are scarce. However, we understand that this may not be clear to the audience when reading our through our discussion. Consequently, we have added a sentence clarifying this position to the start of the 1st paragraph of the Comparison with existing literature subsection. This sentence reads as follows, "*To the author's knowledge, this is the first systematic review of SMRs, so findings are compared with broader MR and deprescribing literature.*"
Tables	
Table 1: Outcomes domain: Why are adverse drug events not included?	This systematic review was primarily focused towards collating evidence on the impact of SMR on physical, functional outcomes etc…and medicines-optimisation outcomes (including adverse drug events) were a secondary objective as listed in the eligibility criteria (Table 1). These were reported in Table 3.
Reviewer 2	
1. The included interventions vary considerably with respect to professional involvement (GPs, pharmacists,	Thank you for this thoughtful comment and we agree that detail regarding whether certain SMR characteristics drove improvements in outcomes or otherwise, would be

multidisciplinary teams), use of decision-support tools, intervention intensity, follow-up duration, and care settings. Although the authors appropriately chose narrative synthesis because of heterogeneity, the discussion would benefit from a more structured exploration of whether certain intervention characteristics were associated with more favorable outcomes. This would help readers understand which components of SMRs may be most promising.	valuable for the reader. Previously, this detail was missing from our Main Findings subsection of the Discussion, although we did touch on this regarding delivery methods (in person vs remote) we did not explore other SMR components. This was mainly due to the small number of studies and their heterogeneity limiting what conclusions we could reliably make. However, we do see the value of including some detail on this, so have added reasonable exploration to the 2nd paragraph and made sure not to overclaim.<br><br>This paragraph now reads: "*Reviews were often conducted by small teams of primary healthcare professionals (including general practitioners, pharmacists or multidisciplinary teams). In general, these were performed in general practice or at the home of the patient, with patients selected for SMRs generally older in age. Although the small number of studies and their heterogeneity preclude formal comparison, improvements clustered by what each study measured rather than by how the review was configured: gains in medicines-optimisation outcomes appeared across GP-led, pharmacist-led and multidisciplinary reviews and in studies with and without decision-support tools, whereas clinical endpoints showed little consistent change in any configuration. Improvements were therefore concentrated in the prescribing changes SMRs directly enact, rather than the broader health outcomes they are ultimately intended to improve. However, the specific delivery methods of the SMRs were not clearly defined in the studies reviewed, limiting the review's ability to draw generalisable conclusions about the effectiveness of different SMR delivery approaches.*"
2. SMRs are presented as person-centred interventions, yet patient experience and patient-reported outcomes receive comparatively little attention in the Results and Discussion sections. The manuscript would benefit from a more explicit synthesis of outcomes related to patient satisfaction, understanding of medicines, shared decision-making, and treatment burden, which are central to the rationale for implementing SMRs. If those are not available, that should be emphasized in the Limitations section.	Thank you again for highlighting this. We agree that patient experience and patient-reported outcomes, being central to the rationale for SMRs, warranted more prominent and explicit synthesis than our original approach (which grouped them under the broader outcome umbrellas) allowed.<br><br>In response, we have made the following changes. We first created a dedicated outcome domain "person-centred outcome" in Table 2 of the results, so these findings are presented clearly and separately rather than being absorbed into other domains. This domain synthesises three studies and is reported as so, "*One study reported that 87.7% of participants felt the review improved their improved understanding of medicines and 30.5% that it reduced the negative impact of medicines on their daily activities.*[47] *Two further studies assessed patient-reported constructs but found no significant between-group effects.*[43,44] *For example, one reported no difference in treatment burden or attitudes to deprescribing*[43] *while the other reported no difference in satisfaction with share decision-making or beliefs about medicines*[44]."

	Second, we added an explicit synthesis of these outcomes to the Main findings subsection of the discussion, noting that the person-centred outcomes were sparsely measured and that the constructs most central to the SMR rationale showed no improvement where assessed. More specifically, we added this sentence to the 1st paragraph, "*Person-centred outcomes were rarely measured, and where treatment burden, shared decision-making and beliefs about medicines were assessed, none improved.*"<br><br>Thirdly, as the comment suggests, we have also emphasised this gap in the Strengths and limitations subsection of the discussion, noting that few of the included studies measured person-centred outcomes, limiting our ability to evaluate whether these interventions achieve their intended aims. Specifically, we add this sentence to the third paragraph: "*Another limitation of the evidence base is that few studies measured person-centred outcomes, which are central to the justification for SMRs. This restricts our ability to assess whether these interventions effectively meet their intended patient-orientated goals.*"<br><br>Plus, we reinforced this in our Implications for research subsection of the discussion, mentioning that: "*If SMRs are intended as holistic health assessments, their impact on physical, mental, and functional health, as well as patient-reported outcomes, requires more rigorous investigation.*"<br><br>Together, these changes ensure that patient experience and patient-reported outcomes are bought to the forefront and that their limited measurement across the evidence base is clearly acknowledged.
3. The conclusions should be interpreted with greater caution. Although the review did not identify consistent improvements in clinical outcomes, the evidence base is limited, heterogeneous and includes several studies judged to be of fair or poor quality. In addition, many of the included studies appear to have been primarily designed to assess prescribing-related outcomes rather than patient-centered clinical endpoints. The authors should therefore discuss more explicitly how these limitations may influence the interpretation of the findings and avoid conclusions that could be interpreted as evidence of lack of effectiveness.	We thank the reviewer for this important point and have revised the manuscript to interpret the findings with greater caution throughout. We have softened language that could be read as evidence of ineffectiveness across the Results, Discussion and Conclusion. Phrases such as "*ineffective*" have been replaced with "*did not consistently improve*", and stronger evaluative terms have also been removed. For example, the intention-impact gap in the Conclusion is now described as an "*apparent gap that warrants attention*" rather than a "*concerning disconnect*".<br><br>The Conclusion now reads: "*SMRs were introduced as a person-centred response to the challenges of polypharmacy, intended to provide a holistic assessment of patients' health and medications. This systematic review found that while SMRs led to improved prescribing practices, they did not significantly enhance endpoint outcomes or reduce healthcare utilisation in patients with multimorbidity and polypharmacy. This*

	should be understood in the context of an evidence base that is small, diverse, and primarily focused on detecting prescribing rather than health outcomes, reflecting both the constraints of the available evidence and potentially the intervention itself. This apparent gap between intention and impact warrants attention, particularly given the comprehensive nature of the ideal SMR model. A key limitation was the lack of clarity around how these SMR interventions were delivered, making it challenging to identify the most effective approaches. Despite these challenges, SMRs offer a promising framework for improving prescribing quality, though their broader person-centred benefits remain to be demonstrated. To fulfil their potential, SMRs must be underpinned by clear policy guidance, embedded within collaborative models of care and supported by adequate training and structural resources. Clinicians need the time and support to deliver meaningful reviews so patients can truly benefit from the intended value of SMRs"<br><br>Plus, we have made explicit that this interpretation is constrained by the nature and quality of the evidence base. In the Main findings, we now note that several included studies were of fair or poor methodological quality and that the strength of our conclusions is correspondingly limited. This can be found in the 2nd sentence of the 1st paragraph and reads, "*The majority, however, were designed to assess prescribing-related outcomes rather than broader measures, and several were of fair or poor methodological quality; thus, the strength of this conclusion may be limited*"<br><br>This has also been expanded upon in the Strengths and limitations section, where we report that study quality was variable. This has been added to the last paragraph and reads as follows, "*Study quality was variable, with several studies rated fair or poor, reflecting common methodological constraints such as small samples, recruitment and selection biases and baseline imbalances. The absence of consistent improvement in clinical outcomes should therefore be interpreted with corresponding caution.*"<br><br>In addition, we have emphasised throughout the Discussion that most included studies were primarily designed to assess prescribing-related outcomes rather than patient-centred or clinical endpoints. We hope that these revisions ensure that our conclusions better describe the current limitations of the evidence rather than a demonstrated lack of effectiveness.

VERSION 2 - REVIEW

Reviewer 1
Name Gangannagaripalli, Jaheeda
Affiliation The University of Manchester, NIHR ARC GM /Healthy Ageing research group, School of Health Sciences
Date 15-Sep-2026
COI

Thanks for making the suggested changes. The draft has improved significantly since the last version. Can the authors add references in the risk of bias section - 'two studies were rated as good quality' but the references are missing

Reviewer 2
Name Divac , Nevena
Affiliation University of Belgrade
Date 07-Sep-2026
COI

The authors have responded seriously and substantively to my concerns. Importantly, they made concrete methodological and interpretative changes throughout the manuscript which is, in my opinion, now significantly improved.
