# Supplementary material for: Structured medication reviews for adults with multimorbidity and polypharmacy in primary care: a systematic review
Source: BMJ Open. 2026 Oct 22;16(10):e117955. doi: 10.1136/bmjopen-2026-117955 (PMC13691817; doi:10.1136/bmjopen-2026-117955)
Supplement: Supplementary file 2 [file bmjopen-16-10-s001.docx]

**Appendix A: Search strategies**

**Table 1:** Search for Embase Classic + Embase and OVID MEDLINE (R) ALL

| # | Query |
| --- | --- |
| 1 | ((Multimorbidity or (multimorbid$ or multi-morbid$) or (multidisease? or multi-disease?) or (multiple adj1 (ill$ or disease? or condition? or syndrom$ or disorder?)) or "multi Comorbidity" or comorbid$ or co-morbid$ or "Multiple Chronic Conditions" or (coocur* or co-ocur* or co-exist* or multiple*)) adj3 (Disease? or ill* or care or condition)).mp.  [mp=ti, ab, hw, tn, ot, dm, mf, dv, kf, fx, dq, bt, nm, ox, px, rx, ui, sy, ux, mx] |
| 2 | (Polypharmacy or "multiple medication*" or "multiple medicine*" or "multiple  drug*").mp. or exp Polypharmacy/ or "many medication*".mp. [mp=ti, ab, hw, tn, ot, dm, mf, dv, kf, fx, dq, bt, nm, ox, px, rx, ui, sy, ux, mx] |
| 3 | ("Primary Health Care" or primaryAND health).mp. or ("Family Practice"/ or "General Practice"/ or "Physicians, Family"/ or "General Practitioners"/ or "Primary Health Care"/) or (((primary or "primary care" or family or general) and (doctor* or physician* or practice* or practitioner* or medicine)) or "family health team*").mp. or (("primary care" or "family practice" or "family health" or family or "general practice") and nurs*).mp. or ("general practice clinic*" or "family practice clinic*" or "primary care clinic*" or "family  clinic*").mp. [mp=ti, ab, hw, tn, ot, dm, mf, dv, kf, fx, dq, bt, nm, ox, px, rx, ui, sy, ux, mx] |
| 4 | ("medical record review" or "medic* review" or "Structured medic* review drug optimi**" or "drug utili*ation review").mp. or exp "Utili*ation Review"/ or exp "Drug Utili*ation Review"/ or ((drug or drugs or medication* or medicine* or pharmaceutic* or pharmacy or prescription* or prescribing) adj (Review* or program* or programme* or assessment* or consult* or care or intervention or management or evaluation*)).mp.  [mp=ti, ab, hw, tn, ot, dm, mf, dv, kf, fx, dq, bt, nm, ox, px, rx, ui, sy, ux, mx] |
| 5 | 1 or 2 |
| 6 | 3 and 4 and 5 |
| 7 | limit 6 to yr="1997 -Current" |

**Table 2:** Web of Science

| # | Query |
| --- | --- |
| 1 | (TS=(Multimorbidity OR multimorbid* OR multi-morbid* OR multidisease* OR multi-disease* OR "multiple" ADJ1 (ill* OR disease* OR condition* OR syndrom* OR disorder*) OR "multi Comorbidity" OR comorbid* OR co-morbid* OR "Multiple Chronic Conditions" OR coocur* OR co-  ocur* OR co-exist* OR multiple*) AND (TS=(Disease* OR ill* OR care OR condition*))) |
| 2 | (TS=(Polypharmacy OR "multiple medication*" OR "multiple medicine*" OR "multiple drug*" OR  "many medication*")) |
| 3 | (TS=("Primary Health Care" OR "primary AND health") OR TS=("Family Practice" OR "General Practice" OR "Physicians, Family" OR "General Practitioners" OR "Primary Health Care") OR TS=((primary OR "primary care" OR family OR general) AND (doctor* OR physician* OR practice* OR practitioner* OR medicine) OR "family health team*") OR TS=("primary care" OR "family practice" OR "family health" OR family OR "general practice") AND TS=(nurs*) OR TS=("general  practice clinic*" OR "family practice clinic*" OR "primary care clinic*" OR "family clinic*")) |
| 4 | (TS=("medical record review" OR "medic* review" OR "Structured medic* review drug optimi**" OR "drug utili*ation review") OR TS=("Utili*ation Review") OR TS=("Drug Utili*ation Review") OR TS=((drug OR drugs OR medication* OR medicine* OR pharmaceutic* OR pharmacy OR prescription* OR prescribing) NEAR/0 (Review* OR program* OR programme* OR assessment*  OR consult* OR care OR intervention OR management OR evaluation*))) |
| 5 | #1 OR #2 |
| 6 | #3 AND #4 AND #5 |
| 7 | Timespan: 1997-01-01 to present day |

**Table 3**: Search for CINAHL

| **#** | **Query** |
| --- | --- |
| 1 | Multimorbidity OR (multimorbid$ OR multi-morbid$) OR (multidisease? OR multi-disease?) OR (multiple ADJ1 (ill$ OR disease? OR condition? OR syndrom$ OR disorder?)) OR "multi Comorbidity" OR comorbid$ OR co-morbid$ OR "Multiple Chronic Conditions" OR (coocur* OR co-  ocur* OR co-exist* OR multiple*)ADJ3 (Disease? OR ill* OR care OR condition) |
| 2 | (Polypharmacy or "multiple medication*" or "multiple medicine*" or "multiple drug*").mp. or exp Polypharmacy/ or "many medication*".mp. [mp=ti, ab, hw, tn, ot, dm, mf, dv, kf, fx, dq, bt, nm, ox, px, rx, an, ui, sy, ux, mx] |
| 3 | ("medical record review" or "medic* review" or "Structured medic* review drug optimi**" or "drug utili*ation review").mp. or exp "Utili*ation Review"/ or exp "Drug Utili*ation Review"/ or ((drug or drugs or medication* or medicine* or pharmaceutic* or pharmacy or prescription* or prescribing) adj (Review* or program* or programme* or assessment* or consult* or care or intervention or management or evaluation*)).mp. [mp=ti, ab, hw, tn, ot, dm, mf, dv, kf, fx, dq, bt, nm, ox, px, rx, an, ui, sy, ux, mx] |
| 4 | ("Primary Health Care" or primaryAND health).mp. or ("Family Practice"/ or "General Practice"/ or "Physicians, Family"/ or "General Practitioners"/ or "Primary Health Care"/) or (((primary or "primary care" or family or general) and (doctor* or physician* or practice* or practitioner* or medicine)) or "family health team*").mp. or (("primary care" or "family practice" or "family health" or family or "general practice") and nurs*).mp. or ("general practice clinic*" or "family practice clinic*" or "primary care clinic*" or "family clinic*").mp. [mp=ti, ab, hw, tn, ot, dm, mf, dv, kf, fx, dq, bt, nm, ox, px, rx, an, ui, sy, ux, mx] |
| 5 | S1 OR S2 |
| 6 | S3 AND S5 |

**Appendix B**

**Table 1:** Study characteristics

| Author, (year) | Country | Study design | Follow-up (mos) | Study participants | Comparator | Intervention | SMR components | | | | | Key outcomes |
| --- | --- | --- | --- | --- | --- | --- | --- | --- | --- | --- | --- | --- |
|  |  |  |  |  |  |  | Patient selection criteria | Setting | HCP | Nr of patient contacts | Mode of delivery |  |
| a) Jungo et al, 2023  b) Jungo et al, 2024 | Switzerland | a) Cluster randomized clinical trial in 43 primary care practices  b) cost-effectiveness analysis | 6, 12 | Median age (IQR) = 77 (73-83)  Median long-term medications = 7 (4-10)  Median comorbidities (IQR) = 7 (4-10) | Usual care  (n=163) | Six-step medication review using STRIPA (web-based electronic clinical decision support system)  n=160 | Age ≥65 years, ≥ 5 long term medications (≥90 days), ≥ 3 chronic conditions | General practice | GP | 1 | Not reported | a) 58.5% of patients had at least one prescribing recommendation implemented. However, at 12 months, there was no improvement observed in MAI, prescribing omissions, or safety outcomes. Nonetheless, fewer safety events were reported in the intervention group compared to the control group at six and 12 months. No significant difference in reducing the number of falls  b) Intervention was dominant, cheaper and more effective than usual care. CHF 1,857 cost saving per participant (95%CI: -3,620 to -93) (p <0.039) QALY gain per participant 0.026 QALYs (95% CI: 0.013 to 0.040) (0.001). Men, patients aged 65-75 years and those aged over 85 years showed stronger effects. |
| Leendertse et al, (2013) | The Netherlands | Open controlled study | 12 | Mean age (95% CI) = intervention; 75.8 (74.9 to 76.4), control; 75.7 (75.1 to 76.7)  Number of prescription = intervention; 7.8 (7.7 to 8.2) control; 7.9 (7.5 to 8.2)  Mean comorbidities (95% CI) = intervention; 5.2 (4.7 to 5.6), control; 3.4 (3.0 to 3.9) | Usual care  (n=310) | Multicomponent pharmaceutical care intervention  (n=364) | Age ≥ 65, ≥ 5 chronically prescribed drugs with ≤ 1 refill rate of < 80% or > 120%. ≥ 1drugs from the Anatomical Therapeutic Chemical Class A or Class B | General practice | GP, pharmacist and primary care practice nurse | ≥3 | Not reported | An average of 2.8 drug therapy problems and 0.7 care issues were identified per patient. 59% of all interventions involved the start of new drug therapy. No statistically significant differences were found in survival, adverse drug events or quality of life, between the intervention and control groups. In the intervention group, 1.6% medication-related hospital admissions were reported, compared to 3.2% in the control group. |
| Lenander et al, (2014) | Sweden | Randomised controlled trial | 12 | Mean age (95% CIs) = intervention; 79.0 (77.6 to 80.4), control;  78.6 (76.8 to 80.3)  Mean number of prescription (95% CI) = intervention; 8.6 (7.8 to 9.3), control; 7.4 (6.6 to 8.2)  Mean diagnosis per patient (95% CI) = intervention; 5.1 (4.7 to 5.5), control; 4.7 (4.2 to 5.2) | Usual care  (n=102) | Preparatory structured pharmaceutical advice  (n=107) | Age ≥ 65, with ≥ 5 medications who were already scheduled for an appointment with a GP | General practice | Geriatric pharmacist | 1 | Not reported | No significant between-group difference in drug-related problems was observed (p=0.72), but there was a significant reduction in the intervention group (1.73 to 1.31 per patient, p<0.02). The reduction in number of drugs was significantly greater in the intervention group (p<0.046). Patients in the intervention group did not experience fewer hospital admissions or fewer days in hospital, and there was no significant difference in primary care utilisation during follow-up compared to the control group. Moreover, self-rated health remained unchanged in the intervention group, while a significant drop was reported in the control group (p<0.047). |
| Mahlknecht et al, (2021) | Italy | Cluster randomized controlled trial | 24 | Median age (IQR) = 81 (78-85)  Number of prescriptions, median (IQR) = 9 (8-11)  Median comorbidities (IQR) = 5 (4-6) | Usual care  (n = 272) | Interdisciplinary review  (n=307) | Age ≥ 75, with ≥ 8 active agens/drugs | Primary care | Specialist in internal medicine, a clinical pharmacolo- gist, and an evidence-based medicine (EBM) expert, GP | 1 | Not reported | The primary composite outcome (non-elective hospitalisation or death at 24 months) did not differ significantly between groups (adjusted OR 1.46, 95% CI 0.99 to 2.18). 15.8% of prescriptions were rated inappropriate; definitive discontinuation was achieved for 67 (16.0%) of the 419 inappropriate drugs and 37.2% of patients had at least one drug stopped. Falls occurred significantly less frequently in the intervention group (adjusted OR 0.55, 95% CI 0.31 to 0.98, p=0.04). No significant differences were found in mortality, fractures, number of drugs, quality of life, affective status or genitiveve function. |
| a) McCarthy et al, (2022)  b) Gillespie et al, 2025 | Ireland | a) Cluster randomised controlled trial  b) cost-effectiveness | 6, 12 | Mean age (SD) = 76.5 (6.83)  Mean number of prescriptions = 17.37  Mean comorbidities (SD, range) = Not reported but multimorbidity defined by greater than 15 repeat medicines | Usual care  (n=196) | GP-led SPPiRE medication review  (n=208) | Age ≥ 65, with ≥ 15 prescribed medications | General practice | GPs | 1 | Face to face | There was a significant reduction observed in the number of medicines and PIP at follow-up. A small but significant effect was noted in the adjusted difference in the number of medicines between the groups. There was no evidence of an effect on the adjusted odds of having a PIP in the intervention group compared to the control. More medicines were stopped in the intervention group, and there was a significant reduction in the odds of being prescribed 15 medicines compared to the control. No evidence of an effect was demonstrated on any PIP-related, patient-reported outcome measures (treatment burden, attitudes to deprescribing, satisfaction with medicines, willingness to stop a medicine and health-related quality of life), or on healthcare utilisation. There was a reduction in the number of GP visits and an increase in the number of telephone consultations in both groups.  b) Intervention was on average cheaper but more effective than usual care  410 euros lower (95% CI -2,211 to +1,409) but this was not statistically significant  +0.014 QALYs (95% CI -0.011 to +0.039) but not statistically significant  Probability cost effective: 99.3% at 20,000 euros/QALY  98.8% at 45,000 euros/QALY |
| Muth et al, (2018) | Germany | Cluster randomised controlled trial | 6, 9 | Mean age (SD) =  72.5 (6.5)  Average of 8 prescriptions taken as 9 single doses a day.  Mean Charlson Comorbidity score (SD) = 3.0 (2.0) | Usual care  (n=253) | Prioritsing multimedication in multimorbidity multicomponent review  (n=252) | Aged ≥60, ≥3 chronic conditions under pharmacological treatment, ≥5 long-term prescriptions of drugs with systemic effects | General practice | GP, HCA | 1 | Not reported | The intervention had no significantly effect on the mean MAI sum scores, which decreased by 0.3 points in the intervention group and 0.8 in the control, leading to a non-significant adjusted mean difference of 0.7 (95% CI -0.2 to 1.6) in favour of the control group at 6 months; the 9 month difference was also non-signficant (MD 0.6, -0.5 to 1.7). There were non-significant changes observed in quality of life (EQ-5D index), expected or desired life duration, functional status (VES-13), chronic pain (von Korff drug/does/regimen scores). The number of prescriptions did not differ significantly, though the adjusted mean number of drugs was 1.0 higher in the intervention group at follow up. Hospital stays and days showed no significant difference. Patient-reported outcomes showed no signficant between-group differences. Satisfcation with shared-decision-making (Man-Son-Hing scale) 9-month MD -0.2 (95% CI -1.0 to 0.5), p=0.519). Beliefs about medicines (BMQ) non-significant throughout. |
| Rieckert et al, (2020) | Austria, Germany, Italy, and the United Kingdom | Cluster randomized controlled trial | 8, 16, 24 | Mean age (SD) = 81.5 (4.4)  Number of prescriptions = 10.5 (2.4)  Mean diagnoses (SD): 9.5 (4.9) | Usual care  (n=1951) | Electronic decision support tool comprising a comprehensive drug review to support general practitioners  (n=1953) | Age ≥ 75, with ≥ 8 prescribed medications | General practice | GP | ≥4 | Not reported | The primary composite outcome (unplanned hospital admission or death by 24 months) did not differ significantly between the groups in the intention-to-treat analysis (OR 0.88, 95% CI 0.73-1.07), though a significant reduction favoring the intervention emerged in the per-protocol analysis (OR 0.82, 0.69-0.98). The number of drugs at follow-up was significantly lower in the intervention group (adjusted mean difference, -0.45, 95% CI -0.63 to -0.26, p<0.001). No significant differences were found in mortality, hospital admissions, falls, fractures or quality of life. |
| Tan et al, (2014) | Australia | Prospective pre-post intervention | 3, 6 | Mean age (SD) = 71.7 (11.2)  Median number of prescription s (IQR), = 11.5 (9,15)  Mean comorbidities (SD, range) = Not reported | Pre-intervention  (n=82) | Pharmacist-led multidisciplinary medication review  (n=62) | Age ≥ 18, with ≥ 5 medications that require therapeutic drug monitoring, using medicines for ≥ 3 medical conditions, recent unplanned | Primary care clinic or home | Pharmacist, GP | 2 | Face to face | The median number of MRPs per patient identified by the practice pharmacist decreased from 2 (IQR 1,4) to 0 (IQR 0,1) at 6 months (p<0.001). 73.5% of MRPs identified at baseline were resolved. Medication adherence improved significantly on both the Morisky (44.1% to 62.7%, p=0.023) and TABS (35.6% to 57.6%, p=0.019) scales. There was no signficant change in self-reported general health (median 3.5 to 3, p=0.627) or in health service use 6 months before versus after the intervention. Patients were satisfied with the consultations. |
| a) Kinahan et al, 2025  b) O’Mahony et al, 2024 | Ireland | a) Prospective pre-post intervention study  b) Cost-benefit analysis | ≤12 weeks (majority 2-6 weeks) | Mean age (SD): 76.0 (9.5)  Mean number of co-morbidities (SD): 6.2 (2.3)  Mean number of medicines (SD): 13.8 (4.7) | Pre (n=1471) | Person-centered medication review Post (n= 1471) | Age ≥50 years living in a residential care setting, or ≥ 10 regular prescribed medications, or a greater risk of medicines-related harm or adults of any age approaching the end of their life due to any cause | General Practice | Pharmacists | 2 | Both – Telephone reviews initially and then face-to-face | A) Mean medicines reduced from 13.8 to 12.3 pre/post  Proportion of patients on ≥10 medicines dropped from 88% to 77%. Mean PC-MAI reduced from 27.1 to 9.8, a reduction of 17.3 per patient (95% CI 15.9 to 18.8), p<0.0001; 64.4% reduction), with 99% of patients improving. Of 1056 polypharmacy indicators identified, 70.7% were resolved; ≥1 was present in 47.1% of patients. Patients reported the review helped their understanding of medicines (87.7%), experience of side effects (63.1%), adherence (36.9%) and impact of medicines on daily activities (30.5%). No significant change in quality of life (EQ-5D-3L index 0.871 to 0.885, p=0.14)  b) The mean reduction in drug costs per review was found to provide an annual cost saving of €376 (95% confidence interval €212–€540) per patient reviewed. Applying this unit cost to the 617 annual reviews gives a total direct cost saving of €231,992 per annum. This results in net cost savings of €288 per review, or €177,696 per annum per pharmacist. Net cost savings of up to €450,000 per annum may be achieved. This service is economically dominant under all modelling and sensitivity analyses performed. |
| Abbreviations: CI; confidence intervals, GPP; general practice pharmacist, GP; general physician, STRIPA; Systematic Tool to Reduce Inappropriate Prescribing Assistant, SD; Standard deviation, GAN; geriatric assessment nurse. PIP; potentially inappropriate prescribing, MRPs; medication related problems | | | | | | | | | | | | |

**APPENDIX C.** Quality assessment of the included studies.

| Author | Study design | Q1 | Q2 | Q3 | Q4 | Q5 | Q6 | Q7 | Q8 | Q9 | Q10 | Q11 | Q12 | Q13 | Q14 |
| --- | --- | --- | --- | --- | --- | --- | --- | --- | --- | --- | --- | --- | --- | --- | --- |
| Jungo et al 2023, | Cluster randomised clinical trial | **✓** | **✓** | **✓** | **X** | **✓** | **✓** (except for MAI scores which was adjusted in sensitivity analysis) | **✓** | **✓** | **✓** | **✓** | **✓** | **✓** | **✓** | **✓** |
| Kinahan et al |  | **✓** | **✓** | **X** | **X** | **X** Not for secondary outcomes | **✓** | **X** Outcomes assessed inconsistently across outcomes | **X** Selection bias – collected PC-MAI and PROMs | **X**  Conviencience sampling for PC-MAI/PROMS/EQ-5D |  |  |  |  |  |
| Leendertse et al, (2013) | Open contolled study | **✓** | **✓** | **X** | **✓** | **✓**(not achieved) | **✓** | **✓ (**for medication-related hospitalisation, too short for morality or Qol) | **X** | **✓** | **✓** | **✓** | **✓** | **✓ (not adjusted for cormorbidity)** | **X** |
| Lenander et al 2014 | Randomised controlled trial | **✓** | NR | NR | **X** | **✓** | **✓** (imbalance in number of drugs) | **X** | **✓** | **✓** | **✓** | **✓** | **✓** | **✓** | **X** |
| Mahlknect et al 2021 | Cluster randomised controlled trial | **✓** | **✓** | **✓** | **✓** | **X** | **X** | **X** | **✓** | **✓** | **✓** | **✓** | **✓** | **✓** | **✓** |
| McCarthy et al 2022 | Cluster randomised controlled trial | **✓** | **X** | **✓** | **X** | **✓** | **✓** | **✓** | **X** | **✓** | **X** | **✓** | **✓** | **✓** | **✓** |
| Muth et al 2018 | Cluster randomised controlled trial | **✓** | **✓** | **✓** | **X** | **✓** | **✓** | **✓** | **✓** | **✓** | **✓** | **✓** | **✓** | **✓** | **✓** |
| Rieckert et al 2020 | Cluster randomised controlled trial | **✓** | **✓** | **✓** | **X** | **X** | **✓** | **✓** | **✓** | **X** | **✓** | **✓** | **✓** | **✓** | **✓** |
| Tan et al 2014 | Prospective pre/post intervention | **✓** | **✓** | **X** | **X** | **✓** | **✓** | **✓** | **X** | **X** | **✓** | **X** | NA | **-** | **-** |

**Questions for the Tool for Quality Assessment (National Heart, Lung and Blood Institute Quality Assessment Tool)**

Observational Cohort and Cross-Sectional Studies

1. Was the research question or objective in this paper clearly stated?

2. Was the study population clearly speciﬁed and deﬁned?

3. Was the participation rate of eligible persons at least 50%?

4. Were all the subjects selected or recruited from the same or similar populations (including the same time)? Were inclusion and exclusion criteria for being in the study prespeciﬁed and applied uniformly to all participants?

5. Was a sample size justiﬁcation, power description, or variance and eﬀect estimates provided?

6. For the analyses in this paper, were the exposure(s) of interest measured prior to the outcome(s) being measured?

7. Was the timeframe suﬃcient so that one could reasonably expect to see an association between exposure and outcome if it existed?

8. For exposures that can vary in amount or level, did the study examine diﬀerent levels of the exposure as related to the outcome (e.g., categories of exposure, or exposure measured as continuous variable)?

9. Were the exposure measures (independent variables) clearly deﬁned, valid, reliable, and implemented consistently across all study participants?

10. Was the exposure(s) assessed more than once over time?

11. Were the outcome measures (dependent variables) clearly deﬁned, valid, reliable, and implemented consistently across all study participants?

12. Were the outcome assessors blinded to the exposure status of participants?

13. Was loss to follow-up amer baseline 20% or less?

14. Were key potential confounding variables measured and adjusted statistically for their impact on the relationship between exposure(s) and outcome(s)?

Controlled Intervention Studies

1. Was the study described as randomized, a randomized trial, a randomized clinical trial, or an RCT?

2. Was the method of randomization adequate (i.e., use of randomly generated assignment)?

3. Was the treatment allocation concealed (so that assignments could not be predicted)?

4. Were study participants and providers blinded to treatment group assignment?

5. Were the people assessing the outcomes blinded to the participants' group assignments?

6. Were the groups similar at baseline on important characteristics that could aﬀect outcomes (e.g., demographics, risk factors, co-morbid conditions)?

7. Was the overall drop-out rate from the study at endpoint 20% or lower of the number allocated to treatment?

8. Was the diﬀerential drop-out rate (between treatment groups) at endpoint 15 percentage points or lower?

9. Was there high adherence to the intervention protocols for each treatment group?

10. Were other interventions avoided or similar in the groups (e.g., similar background treatments)?

11. Were outcomes assessed using valid and reliable measures, implemented consistently across all study participants?

12. Did the authors report that the sample size was suﬃciently large to be able to detect a diﬀerence in the main outcome between groups with at least 80% power?

13. Were outcomes reported or subgroups analyzed prespeciﬁed (i.e., identiﬁed before analyses were conducted)?

14. Were all randomized participants analyzed in the group to which they were originally assigned, i.e., did they use an intention-to-treat analysis?

Pre post studies

1. Was the study question or objective clearly stated?
2. Were eligibility/selection criteria for the study population prespecified and clearly described?
3. Were the participants in the study representative of those who would be eligible for the test/service/intervention in the general or clinical population of interest?
4. Were all eligible participants that met the prespecified entry criteria enrolled?
5. Was the sample size sufficiently large to provide confidence in the findings?
6. Was the test/service/intervention clearly described and delivered consistently across the study population?
7. Were the outcome measures prespecified, clearly defined, valid, reliable, and assessed consistently across all study participants?
8. Were the people assessing the outcomes blinded to the participants' exposures/interventions?
9. Was the loss to follow-up after baseline 20% or less? Were those lost to follow-up accounted for in the analysis?
10. Did the statistical methods examine changes in outcome measures from before to after the intervention? Were statistical tests done that provided p values for the pre-to-post changes?
11. Were outcome measures of interest taken multiple times before the intervention and multiple times after the intervention (i.e., did they use an interrupted time-series design)?
12. If the intervention was conducted at a group level (e.g., a whole hospital, a community, etc.) did the statistical analysis take into account the use of individual-level data to determine effects at the group level?

**Table S3.** Quality assessment of the included cost-effectiveness studies.

| Author | Q1 | Q2 | Q3 | Q4 | Q5 | Q6 | Q7 | Q8 | Q9 | Q10 | Q11 | Q12 | Q13 | Q14 | Q15 | Q16 | Q17 | Q18 | Q19 |
| --- | --- | --- | --- | --- | --- | --- | --- | --- | --- | --- | --- | --- | --- | --- | --- | --- | --- | --- | --- |
| Gillespie et al 2025 | **✓** | **✓** | **✓** | **✓** | **✓** 6 month horizon too short for multimorbidity | **✓** | **✓**other medication costs, communite care and private/OOP costs not included | **✓** | **✓**  Marginal costing of med/started/stopped produced negative total costs | **✓** | **✓**  Non-responses for patient reported outcomes | **✓** | **✓** | **NA** | **✓** | **✓** | **X**  No external validity analysis | **✓** | **Partial**  Distributional and equity impacts not formally analyses |
| Jungo et al, 2024 | **✓** | **✓** | **✓** | **✓** | **✓** 12 month horizon matches trial follow up but this may be too short | **✓** | **✓** | **✓** | **✓** | **✓** | **✓** | **✓** | **✓** | **NA** | **✓** | **✓** | **✓** | **✓** | **✓** |
| O’Mahony et al 2024 | **✓** | **X**Comparator not described beyond pre-ewview | **✓** | **Partial**  No controlled counterfactual | **✓**  Horizon may miss longer term effects beyond medication-cost changes | **✓** | **✓** | **✓** | **✓** | **✓** | **✓**  Measurement relies partly on assumption | **✓** | **✓**  Single arm | **X** | **NA** | **X**  Missing probabilistic analysis across all inputs | **✓** | **X**  No external validity analysis | **Partial**  No distributional or equity analysis |

1. Is the study population clearly described?
2. Are competing alternatives clearly described?
3. Is a well-defined research question posed in answerable form?
4. Is the economic study design appropriate to the stated objective?
5. Is the chosen time horizon appropriate to include relevant costs and consequences?
6. Is the actual perspective chosen appropriate?
7. Are all important and relevant costs for each alternative identified?
8. Are all costs measured appropriately in physical units?
9. Are costs valued appropriately?
10. Are all important and relevant outcomes for each alternative identified?
11. Are all outcomes measured appropriately?
12. Are outcomes valued appropriately?
13. Is an incremental analysis of costs and outcomes of alternatives performed?
14. Are all future costs and outcomes discounted properly?
15. Are all important variables, whose values are uncertain, appropriately subjected to sensitivity analysis?
16. Do the conclusions follow from the data reported?
17. Does the study discuss the generalizability of the results to other settings and patient/client groups?
18. Does the article indicate that there is no potential conflict of interest of study researcher(s) and funder(s)?
19. Are ethical and distributional issues discussed appropriately?
